# Microalgal Lipid Extracts Have Potential to Modulate the Inflammatory Response: A Critical Review

**DOI:** 10.3390/ijms22189825

**Published:** 2021-09-11

**Authors:** Tiago Alexandre Conde, Ioannis Zabetakis, Alexandros Tsoupras, Isabel Medina, Margarida Costa, Joana Silva, Bruno Neves, Pedro Domingues, M. Rosário Domingues

**Affiliations:** 1 CESAM-Centre for Environmental and Marine Studies, Department of Chemistry, Santiago University Campus, University of Aveiro, 3810-193 Aveiro, Portugal; tiagoalexandreconde@ua.pt; 2 Mass Spectrometry Centre, LAQV REQUIMTE, Department of Chemistry, Santiago University Campus, University of Aveiro, 3810-193 Aveiro, Portugal; p.domingues@ua.pt; 3 Department of Medical Sciences, Institute of Biomedicine–iBiMED, University of Aveiro, 3810-193 Aveiro, Portugal; bruno.neves@ua.pt; 4 Department of Biological Sciences, University of Limerick, V94 T9PX Limerick, Ireland; ioannis.zabetakis@ul.ie (I.Z.); alexandros.tsoupras@ul.ie (A.T.); 5 Health Research Institute (HRI), University of Limerick, V94 T9PX Limerick, Ireland; 6 Bernal Institute, University of Limerick, V94 T9PX Limerick, Ireland; 7 Instituto de Investigaciones Marinas-Consejo Superior de Investigaciones Científicas (IIM-CSIC), Eduardo Cabello 6, E-36208 Vigo, Spain; medina@iim.csic.es; 8 R&D Department, Allmicroalgae Natural Products SAA, Rua 25 de Abril 1974, 2445-287 Pataias, Portugal; costa.anamarg@gmail.com (M.C.); joana.g.silva@allmicroalgae.com (J.S.)

**Keywords:** microalgae, lipids, glycolipids, phospholipids, inflammation, anti-inflammatory

## Abstract

Noncommunicable diseases (NCD) and age-associated diseases (AAD) are some of the gravest health concerns worldwide, accounting for up to 70% of total deaths globally. NCD and AAD, such as diabetes, obesity, cardiovascular disease, and cancer, are associated with low-grade chronic inflammation and poor dietary habits. Modulation of the inflammatory status through dietary components is a very appellative approach to fight these diseases and is supported by increasing evidence of natural and dietary components with strong anti-inflammatory activities. The consumption of bioactive lipids has a positive impact on preventing chronic inflammation and consequently NCD and AAD. Thus, new sources of bioactive lipids have been sought out. Microalgae are rich sources of bioactive lipids such as omega-6 and -3 polyunsaturated fatty acids (PUFA) and polar lipids with associated anti-inflammatory activity. PUFAs are enzymatically and non-enzymatically catalyzed to oxylipins and have a significant role in anti and pro-resolving inflammatory responses. Therefore, a large and rapidly growing body of research has been conducted in vivo and in vitro, investigating the potential anti-inflammatory activities of microalgae lipids. This review sought to summarize and critically analyze recent evidence of the anti-inflammatory potential of microalgae lipids and their possible use to prevent or mitigate chronic inflammation.

## 1. Introduction

Noncommunicable diseases (NCD) are disorders that are not transmissible from one person to another (e.g., obesity, diabetes, cancer, autoimmune diseases, cardiovascular diseases, respiratory, and musculoskeletal disorders) and are the result of a multifactorial combination of unhealthy lifestyle habits and genetic predisposition [1]. They are among the leading causes of death, representing around 70% of total global deaths, and are recognized as one of the biggest challenges by the World Health Organization (WHO) and the Food and Agriculture Organization of the United Nations (FAO) [2,3]. One of the leading causes of NCDs is malnutrition, which results from states of obesity, other dietary factors, and undernutrition (e.g., underweight, deficiencies in vitamins, stunting). On the other hand, ageing impacts biological and physiological functions, which promotes and aggravates the development of age-associated diseases (AAD) such as atherosclerosis, hypertension, cardiovascular diseases, Alzheimer’s disease, dementia, arthritis, and osteoporosis [4]. Malnutrition also contributes to the onset of AADs and is often underdiagnosed in elderly patients [5]. Therefore, intervention in dietary habits is a critical approach to tackle these challenges. Another issue associated with unhealthy dietary practices arises from the overexploitation and consequent pollution associated, for example, with current food production, which accounts for 20–35% global greenhouse emissions [3]. A dietary shift to intake small amounts of calories from animal sources and increase the consumption of sustainable, nutrient-rich, and calorically efficient products, such as algae, are recommended by the FAO and WHO to prevent the aggravation of these issues [3].

Most NCDs are associated with low-grade chronic inflammation, characterized by persistently elevated levels of circulating pro-inflammatory cytokines, chemokines, and acute inflammation phase proteins [6]. The persistency of this low-grade chronic inflammation results in cellular, tissue, and organ damage over time, mainly through continuous oxidative stress, which eventually impairs proper body function. Mitigating the inflammatory response can prevent or decrease the severity of NCDs [1]. On the other hand, AADs are associated with the increasing senescence processes associated with ageing, such as telomerase erosion, the oxidative damage of DNA and proteins, mitochondrial dysfunction, oncogene overexpression, or epigenetic factors, that result in stem cell exhaustion, the dysfunction of body systems, and chronic inflammation [7]. To an extent, both NCD and AAD can be prevented by modifying lifestyle-related risk factors, where unhealthy diets play a significant role [8,9]. The modulation of chronic inflammation through diet plays an important role in decreasing the risks and prevalence of NCDs and AADs, as diet can provide components such as omega-3 (ω-3) fatty acids, flavonoids, and vitamins, which can suppress inflammation, decrease oxidative damage, and modulate gene expression [10].

**Figure 1:**
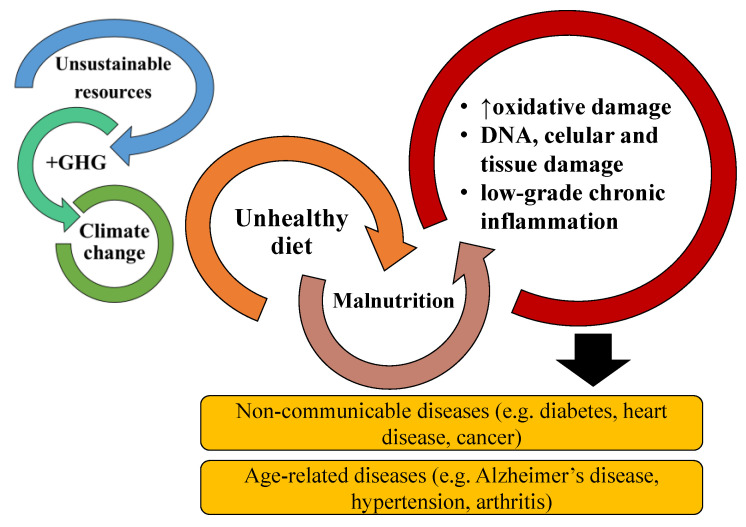
The relationship and risk of noncommunicable diseases and age-associated diseases are increased by malnutrition originating from poor and unhealthy diets. Malnutrition promotes increased oxidative stress, DNA, cellular and tissue damage, and chronic inflammation. Chronic inflammation is associated with most NCDs and AADs. Bad diets are also associated with the unsustainable exploitation of resources, greenhouse gases (GHG) emissions, and impacts on climate change.

The transition to more sustainable and healthy diets that are capable of preventing the development of NCDs and AADs, requires investment in and development of new food sources and ingredients with high nutritional value and diversity with sustainable production and reduced environmental impact (Figure 1) [3]. Microalgae are a sustainable and highly nutritious alternative that could enrich and contribute to the transition to more environmentally friendly and healthy diets [11,12]. They are a diverse and rich source of nutritional components, such as vitamins (e.g., vitamin B12), carbohydrates, proteins, nucleic acids, lipids (e.g., ω-3 polyunsaturated fatty acids (PUFA), others), and functional components (e.g., chlorophylls, which have antioxidant activity) [13]. Their lipid nutritional value is comparable to that of fish oils, offering an alternative to this commonly used source of ω-3 FA [14,15]. Microalgae are considered a promising source of bioactive components, including bioactive lipids such as ω-3 lipids, essential precursors of anti-inflammatory eicosanoids, and polar lipids [11]. Recent research suggests that marine polar lipids as phospholipids or glycolipids, which are highly concentrated in ω-3 PUFAs, could be more effectively delivered than triglycerides in terms of human health. Polar lipids are more stable and have higher bioavailability than triglycerides [16]. Moreover, as a relevant fact, phospholipids have recently been suggested as an excellent vector of DHA and oxygenated DHA metabolites as protectins, enhancing their in vivo role as inflammation resolvers. Among these, glycolipids (GL) have shown chemotherapeutic potential, anti-proliferative effects, potent inhibition of nitric oxide (NO) release, and anti-inflammatory potential [17]. Only a handful of microalgae species are approved for food consumption; however, the use of microalgae and their lipid extracts as food ingredients are recognized as having the potential to prevent NCDs and AADs and to enrich nutrient-deficient diets.

On the other hand, the anti-inflammatory and antioxidant potential of microalgae lipid extracts were intensively studied in the past decade. In this context, this review will address the anti-inflammatory potential of bioactive raw extracts, lipidic fractions, and isolated lipids from microalgae. This information will help valorize and promote microalgal lipids as sustainable and healthy dietary approaches to tackle global malnourishment, NCD, AAD, and environmental degradation.

### Literature Reviewing Strategy

This revision was performed using the platform Web of Science. The keywords used were a combination of “microalgae”, with “anti-inflammatory”, “immunomodulatory”, and immunomodulation. The inclusion criteria were the use of microalgae organic extracts or the use of microalgae isolated lipid classes and species that have been reported to have anti-inflammatory/pro-resolving effects. Out of 138 results, only 32 met the proposed criteria. The excluded works referred to the use of aqueous extracts. Other banned works involved evaluating the anti-inflammatory activity of pigments or other non-lipidic-based molecules that did not fit the purpose of this review.

**Figure 2:**
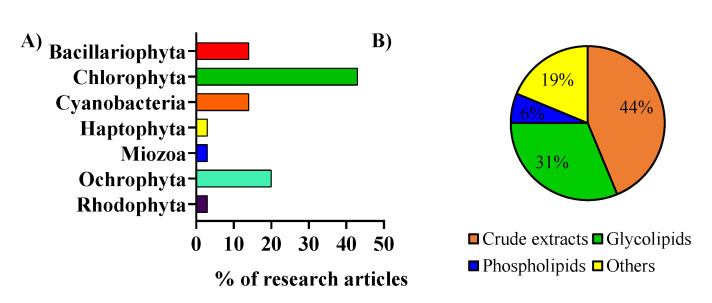
(**A**) Microalgae of different phyla were used in the assays to evaluate the anti-inflammatory activity of lipids. (**B**) Type of lipid extracts from microalgae used to assay the anti-inflammatory activity.

The included works were analyzed and were categorized by considering the microalga phylum (Figure 2A) and the type of lipid extracts or fractions that were used (Figure 2B). Microalgae from the phylum Chlorophyta were the most studied regarding anti-inflammatory activity. Most of the studies used crude lipid extracts in the anti-inflammatory activity assays [18,19,20]. In contrast, others used lipid fractions in specific classes of lipids, such as glycolipids, phospholipids, or others (Figure 2B) [21,22]. Among these, the ones that used glycolipids from microalgae enriched fractions were the most studied [23,24,25].

## 2. Microalgae Lipids: Structural Diversity and Functionality

The microalgae lipidome includes two main groups of lipids: neutral lipids (fatty acids, TAG, and sterols) and polar lipids (sphingolipids, phospholipids (PL), glycolipids (GL), and betaine lipids (BL)). Polar lipids can develop several biological functions, acting as the structural components of cell membranes, which constitute lipoproteins, energy reserves, and signaling molecules [26].

**Figure 3:**
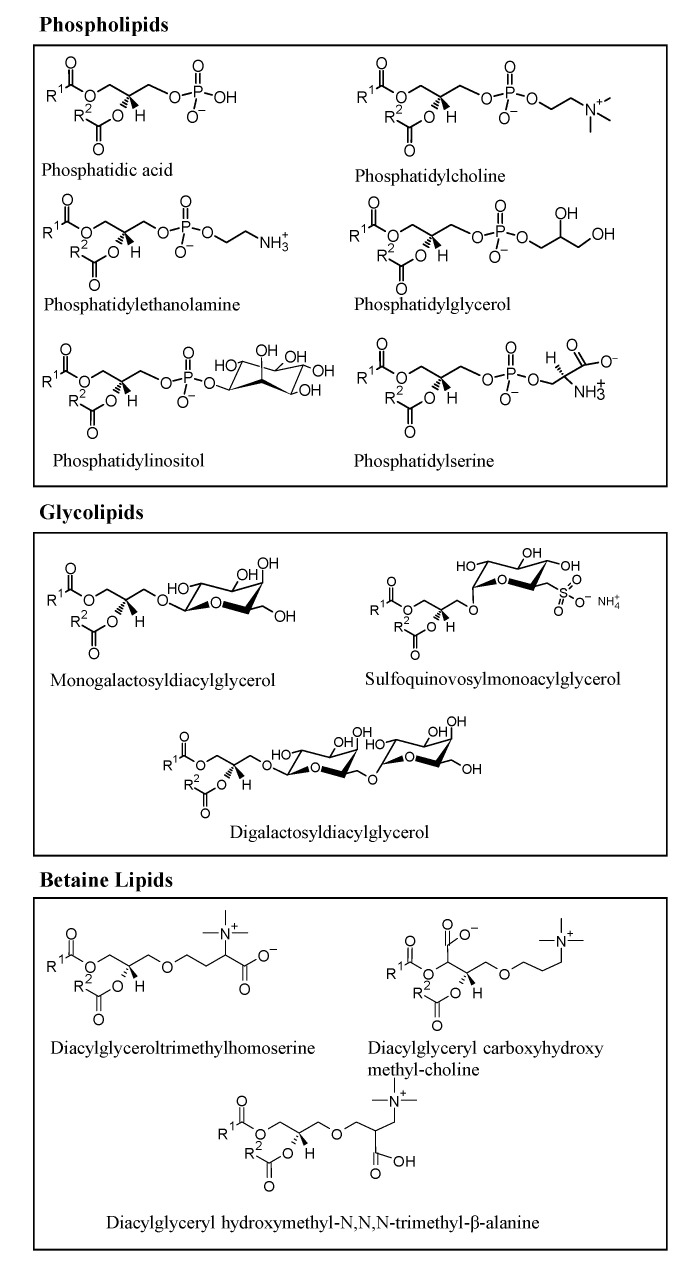
Main classes of polar lipids found in microalgae: glycerophospholipids (or phospholipids), glycoglycerolipids (or glycolipids), and betaine lipids.

Microalgae are rich in polar lipids, constituting 41–92% of total lipids, while neutral lipids constitute 5–51%. The lipidomic profile of several microalgae species have been studied, and significant polar lipids, represented in Figure 3, that have been identified include species from several classes of phospholipids, such as phosphatidic acid (PA), phosphatidylserine (PS), phosphatidylethanolamine (PE), phosphatidylinositol (PI), phosphatidylcholine (PC), phosphatidylglycerol (PG); glycolipids, such as digalactosyldiacylglycerol (DGDG), sulfoquinovosylmonoacylglycerol (SQDG), monogalactosyldiacylglycerol (MGDG); and betaines lipids, such as diacylglyceroltrimethylhomoserine (DGTS), diacylglyceryl hydroxymethyl-N,N,N-trimethyl-β-alanine (DGTA), and diacylglyceryl carboxyhydroxy methyl-choline (DGCC), as represented in Figure 3. Some of the polar lipid species described here were reported as being biologically active [27,28,29,30,31,32,33].

Polar lipids and neutral lipids are the leading FA carriers, which are the most studied lipids in microalgae. [34,35,36,37,38]. Microalgae FAs are typically 12 to 22 carbons long with up to 6 unsaturations; however, short-chain FA and oxidized PUFAs have been reported [39,40]. FAs such as the omega-6 and arachidonic acid (ARA) and the ω-3 FAs, such as α-linolenic acid (ALA), eicosapentaenoic acid (EPA), and docosahexaenoic acid (DHA), are usually associated with the nutritional value and bioactive properties from algae. They are precursors of pro- and anti-inflammatory eicosanoids, respectively, and contribute to the balance of both omega-6 and ω-3 FA, which are important for the normal functioning of the immune system [41,42,43].

Polar lipids have been described as potent bioactive compounds [44,45], and therefore, the interest in understanding their bioactive mechanisms and the extent of their bioactive potential have increased. Several studies have pointed to PL and GL, especially those esterified with ω-3 PUFAs, as possessing antioxidant, anti-inflammatory, anti-obesity, anti-tumor, anti-viral and anti-bacterial activity [16,17,46,47]. The bioactivities observed for these lipids hold exciting potential for natural sources prospection and can pose an alternative to the current sources of these lipids (e.g., fish oils). Fish oil is a commercially available ingredient and nutritional supplement rich in ω-3 PUFAs [3,48,49]. Still, microalgae are considered a sustainable alternative to fish oils and as a source of healthy and bioactive ω-3 PUFAs.

Anti-inflammatory activity was one of the main biological activities reported for microalgae lipid extracts [17]. Moreover, some microalgae polar lipids have been reported to have anti-inflammatory activity [17,22,50]. The following section will discuss the anti-inflammatory activity observed for microalgae lipid extracts and fractionated lipids. These studies evaluated pro-inflammatory markers, and some tried to understand a possible relationship between the selected microalgae species, extracts, and fractions and their immunomodulatory activity. Distinct parameters such as the induction or attenuation of cytokine production, the gene expression of inflammatory markers, and the activation or inhibition of signaling pathways were approached.

## 3. The Anti-Inflammatory Potential of Microalgal Lipid Extracts

The anti-inflammatory properties of bioactive lipids were evaluated using crude extracts from several microalgae, namely Chlorella vulgaris, Chlorella ovalis, Nannochloropsis oculata, Nannochloropsis granulata, Nannochloropsis oceanica, Phaeoductylum tricornutum, Amphidinium carterae; the diatoms Odontella mobiliensis, Pseudonitzschia pseudodelicatissima, Coscinodiscus actinocyclus, and Alexandrium minutum; and the mutant microalgae species Tetraselmis sp. (IMP3 and CTP4) or cyanobacteria Arthrospira maxima [19,32,33,50,51,52,53,54,55]. Different solvents and solvent systems were used to extract the lipids, making an accurate comparison of the results difficult. In these studies, the anti-inflammatory potential was accessed through the evaluation of the release/production of inflammatory mediators, such as the tumor necrosis factor α (TNF-α) and interleukin-6 (IL-6), prostaglandin E2 (PGE2) and NO, and the expression of the key enzymes, such as cyclooxygenase-2 (COX-2) and iNOS. Most of these studies were performed using primary monocytes and lymphocytes or cell lines such as THP-1 and RAW264.7 macrophages, which evaluated the capacity of the test compounds to attenuate or inhibit the lipopolysaccharide (LPS)-induced production of those inflammatory mediators. The different studies reviewed herein can be found described in detail in the Supplementary Table S1.

Very few studies were performed in animal models [23,24,56,57]. The reduction of croton-induced oedema in mice and neutrophils concentration in the wound region of zebrafish was observed when treated with lipids from microalgae [23,24]. Other studies observed the response of pro-inflammatory markers, namely cytokines, to microalgae extracted lipids in inflammatory disease models of induced colitis in rats and diabetic mice [56,57]. The lack of studies performed in animal models of disease hampers elucidation of the complete impact of lipids in the complex network of inflammation.

Details about the studies evaluating the anti-inflammatory effects of microalgal lipids will be described in detail along with the following four different subsections. The subsections will focus on the results gathered in the evaluation of the effect of microalgal lipid extracts on the expression and activity of in vitro key enzymes (Section 3.1**.**), cytokines (Section 3.2**.**), transcriptomic studies (Section 3.3**.**), and other observations from in vitro assays using cells and animal models of inflammatory disease (Section 3.4**.**). Regarding the contribution of lipids to anti-inflammatory potential, most of the data reported so far has focused on the use of microalgal complex extracts, lipidic fractions, or of very few single isolated lipids.

### 3.1. In Vitro Evaluation of Microalgal Lipids Impact in Key Pro-Inflammatory Enzymes

The evaluation of COX-2 and iNOS activity and expression are the most common parameters measured in screenings for anti-inflammatory potential, both generally and for microalgal extracts. COX-2 is an enzyme induced by pro-inflammatory mediators that converts arachidonic acid to prostaglandins. The modulation of its activity and expression provides information regarding the production of pro-inflammatory prostaglandins with a critical role in the initial onset of inflammation [58]. COX-2 inhibition was reported in studies using lipid extracts from *Gloeothece* sp., *Chlorella vulgaris* grown under auto- and heterotrophic conditions, *Chlorococcum amblystomatis* and lipid extracts enriched in ω-3 PUFAs from *Tetraselmis* sp. mutant strains (IMP3 and CTP4)*, Skeletonema* sp., and *Nitzschia palea* [19,53,59,60,61]. These studies evaluated the inhibitory COX-2 activity of the crude lipids with a commercial assay in chemico, as described in Supplementary Table S1. The evaluation of the capacity of lipid extracts to modulate COX-2 activity was also performed in in vitro cells studies, using lipid extracts from *Chlorella vulgaris, Chloromonas reticulata, Micractinium* sp., *Nannochloropsis oculata, Nitzschia palea,* and *Phaeodactylum tricornutum*. The results showed COX-2 inhibition and the downregulation of COX-2 protein levels in Raw264.7 cells [20,52,60,62,63,64,65].

On the other hand, assessing iNOS activity and expression provides information regarding the production of NO, a key molecule for induction and inflammation maintenance. The most common strategy to assess the impact of the tested lipid extracts on iNOS activity relies on evaluating their effects on LPS-triggered NO production by Raw 264.7 macrophages (Supplementary Table S1**)**. For instance, *Amphidinium carterae*, *Chlorella* sp., *Chloromonas reticulata*, *Micractinium* sp., *Nannochloropsis oculata*, *Nitzschia palea,* and *Phaeoductylum tricornutum* lipid extracts were shown to reduce NO levels and iNOS expression induced by LPS in Raw264.7 cells [20,51,52,60,62,63,64,65,66]. Moreover, MGDG and DGDG [67], two classes of glycolipids, and DGTS [22], one of the betaine lipids, extracted from *Nannochloropsis granulata*, revealed intense NO inhibitory activity in Raw264.7 macrophages. Finally, rats supplemented with *Nannochloropsis oculata* extracted glycolipid-rich oil revealed a significant reduction of NO production through the downregulation of iNOS [68].

Abu-Serie et al. [63] reported that extracts from *Chlorella vulgaris* reduced NF-κB expression together with iNOS and COX-2 in an in vitro model of lipopolysaccharide-stimulated white blood cells. The NF-κB signaling pathway plays a central role in inflammation by regulating the transcription of pro-inflammatory genes such as COX-2, iNOS, and multiple pro-inflammatory cytokines [69]. Therefore, the decreased expression of COX-2 and iNOS caused by microalgae lipid extracts could result from the downregulation of this particular pathway, opening new target possibilities that require further study.

### 3.2. In Vitro Evaluation of Microalgae Lipids Impact in Pro-Inflammatory Cytokines

Another essential step to determine the anti-inflammatory potential of microalgae lipid extracts is evaluating the production/release of pro-inflammatory cytokines, such as TNF-α, IL-1β, and IL-6, in activated immune cells. Pro-inflammatory cytokines are predominantly produced by activated immune cells (e.g., macrophages, monocytes, lymphocytes) and play an essential role in pro-inflammatory reactions [70]. Their inhibition results in the attenuation of the inflammatory response, posing a decisive step towards inflammation resolution. The assessment of TNF-α, IL-1β, and IL-6 levels in cell lines (Raw264.7 and THP-1 macrophages) reported for lipid extracts from *Nitzschia palea*, *Chlorella vulgaris*, *Tetraselmis suecica*, *Micractinium* sp., *Aurantiochytrium mangrovei*, *Phaeodactylum tricornutum*, *Chloromona reticulata,* and *Spirulina maxima* showed the capacity to downregulate their production [20,60,62,63,64,65,66,71]. When compared to the use of crude extracts to evaluate the downregulatory effect of microalgae lipids on pro-inflammatory cytokines, the studies using isolated lipid classes and species are reduced. Oxylipins resulting from the enzymatical oxidation of PUFAs, isolated from *Chlamydomonas debaryana* and *Nannochloropsis gaditana*, and LPC(16:0), isolated from *Cylindrotheca Closterium,* showed the capacity to downregulate LPS-triggered TNF-α production in THP-1 macrophages [72,73]. The induction of IL-6 in LPS-activated Raw264.7 cells was decreased with free and esterified DGLA from a mutant strain of the microalga *Lobosphaera incisa* P127 [74]. Another study observed a reduction in TNF-α, IL-6, and IL-1β expression in peritoneal blood mononuclear cells (PBMC) treated with ergosterol and 7-dehydroporiferasterol isolated from the microalga *Dunaliella tertiolecta* [75]. This mix of phytosterols from the microalga *Dunaliella tertiolecta* raised anti-inflammatory cytokine IL-10 levels, strengthening the anti-inflammatory potential. Ávila-Román et al. described newly isolated oxylipins from *Chlamydomonas debaryana* derived from 16:4 and 18:4 fatty acids and used them as diet supplementation in a 2,4,6-trinitrobenzenesulfonic acid (TNBS)-induced colitis animal model, observing a downregulation of TNF-α. The most active oxylipin was a C-16 hydroxy acid [72].

### 3.3. Transcriptomic Analysis to Evaluate Anti-Inflammatory Potential of Microalgae Lipids

Transcriptomic analysis presents a more complex technique that offers more data regarding which proteins, enzymes, cytokines, and chemokines are up- or down-regulated in immune cells incubated with microalgae lipids [76]. To the extent of our knowledge, only Maria et al. has performed transcriptomic analysis aiming to study the effect of microalgal lipids on several pro-inflammatory RNA transcripts from immune cells activated with LPS [77]. Robertson et al. isolated glycolipid fractions rich in SQDG, MGDG, and DGDG from the microalga *Pavlova lutheri* and used them to treat LPS activated THP-1 derived macrophages. Transcriptomic analysis of the cells revealed the downregulation of multiple pro-inflammatory genes: *TLR1, TLR2, TLR4, TLR8, TRAF5, TRAF6, TNFSF18, IL6R, IL23, CCR1, CCR4, CCL17, STAT3, and MAP3K1.* Additionally, the authors observed that these glycolipids inhibited the LPS-induced pro-inflammatory Toll-like receptor (TLR) and NF-κB signaling pathways, which were correlated with the observed downregulation of several pro-inflammatory mediators mentioned in the previous subsection [69].

### 3.4. Other Markers of Attenuation of the Inflammatory Response

A few studies have explored the impact of lipids extracted from microalgae on cells or animal models of inflammatory diseases and injury. Sulfolipids isolated from the microalga *Porphyridium cuentum* inhibited the generation of the superoxide anion in peritoneal leukocytes primed with phorbol myristate acetate [21].

Glycolipid-rich extracts with gamma-linolenic acid isolated from *Spirulina platensis* reduced neutrophil gathering in the wound region in a model of injured zebrafish [23]. Other MGDG, DGDG, and SQDG-rich extracts attenuated croton–oil-induced oedema in a mice model [24]. An isolated betaine lipid, MGTS(20:5), was responsible for the inhibition of LDL oxidation through the increased expression of paraoxonase 1 (PON1) in J-774A macrophages [78,79].

In addition, microalgae bioactive lipids from *Spirulina* and *Chlorococcum sp.* have also been found to inhibit both PAF and thrombin-induced aggregation of platelets [32,33]. The anti-inflammatory and anti-thrombotic potency of *Spirulina* bioactive lipid extracts and protein, polysaccharides, and lipids from *Chlorococcum sp.* were evaluated against the thrombo-inflammatory pathways induced by potent inflammatory and thrombotic mediators such as platelet-activating factor (PAF) and thrombin in ‘platelets’ disease models from rabbits or humans, respectively [32,33]. *Spirulina*-derived lipid bioactives were found to potently inhibit rabbit platelet aggregation induced by PAF or thrombin with phycocyanobilin and specific polar lipids, such as SQDG and PC fractions [32].

Similarly, *Chlorococcum sp.* bioactive lipids were used to assess the potential anti-aggregation properties in human platelets. Again, the polar lipids (phospho- and glycolipids) were the most bioactive against both PAF and thrombin-induced human platelet aggregation [33]. Dereplication, by LC-MS-based lipidomics, revealed that the most bioactive polar lipids were SQDC, MGDG, DGDG, PC, and PE baring either oleic acid (MUFA) or the essential ω-3 PUFA alpha-linolenic acid (ALA) within their structures (at the sn2 position of their glycerol backbone).

Such dietary polar lipids bioactives, and especially those of marine origin, have been found to develop direct antagonistic effects against PAF-binding on the specific cell membrane receptor for PAF (PAF-Receptor) or to indirectly affect its phospholipid microenvironment in the cell membranes. These lipids can also inhibit PAF-synthesis and thus reduce blood PAF levels to homeostatic ones, with several anti-inflammatory, anti-tumor, and anti-atherogenic cardioprotective health benefits [47,80].

PL bioactives are also a good source of anti-inflammatory MUFAs (oleic acid) and ω-3 PUFAs (ALA), as their bio-functional fatty acids have high bioavailability due to their amphiphilic properties [16]. Moreover, when such fatty acids are released by specific cytoplasmic phospholipases A2 (PLA2) from such bioactive PLs located in cell membranes and/or lipoproteins, they further facilitate the production of anti-inflammatory eicosanoids. Those compounds act antagonistically to other inflammatory and thrombotic eicosanoids (prostaglandins, leukotrienes, and thromboxanes), which are usually produced by n-6 PUFAs such as arachidonic acid. The latter further supports the health benefits derived from marine PL bioactives rich in MUFAs and ω-3 PUFAs, namely preventing cardiovascular diseases [81,82].

Overall, multiple studies have reported anti-inflammatory activity with different lipids and different microalgae species (Supplementary Table S1). To understand their dynamics as potent bioactives, it is necessary to understand the interplay between activity and microalgae lipids. The following section intends to shed some light on these dynamics.

## 4. Interplay between Activity and Microalgae Lipids

Several studies have evaluated the association of few lipid classes with anti-inflammatory activities, such as oxylipins, ω-3 fatty acids, and polar lipids (Figure 3). Moreover, some of these lipids are already regarded for their involvement in inflammation [83]. Oxylipins derived from the ω-3 PUFAs are considered potent anti-inflammatory compounds [84]. Two of the studies mentioned above have reported the presence of anti-inflammatory oxylipins in microalgae derived from C16 and C18 PUFAs (e.g., (13S)-HOTE) and are associated with the suppression of TNF-α, iNOS, and COX-2 expression and activity [56,72]. On the other hand, ω-3 fatty acids, such as EPA and DHA, are essential regulators of the inflammatory process, as they can compete with arachidonic acid to produce eicosanoids (i.e., prostaglandins, lipoxins, leukotrienes, resolvins, thromboxanes), with anti-inflammatory anti-aggregatory and vasodilatory effects properties [85,86]. These molecules directly or indirectly reduce the activity of nuclear transcription factors, such as NF-κB, and consequently suppress the production of pro-inflammatory mediators, such as COX-2, TNF-α, and IL-1β [87]. Evidence relating to the downregulation of pro-inflammatory mediators such as cytokines (TNF-α, IL-1β, IL-6), transcription factors (NF-κB, MAPK, ERK) or receptors (TLR4) by ω-3 FA alone are also in the literature [88]. However, the relationship between ω-3 FA structure and this downregulatory activity is far from elucidated. The generation of oxylipins remains the focus, considering their established roles in cell–cell communication and as mediators in inflammation and pathophysiologic events [84]. Cytokines lead a significant multilevel impact on the regulation of enzymes in the individual oxylipin pathways and on the generation of the lipid mediators essential for their action [89]. Cell activation is accompanied by the remodeling of the membrane components that appear to be crucial in signal transduction [90]. Changes in the fatty acid composition of cell membranes lead to the enzymatic competition of desaturases, LOX, and COX enzymes for fatty acids and provoke the specific generation of oxylipins [91].

Polar lipids, such as phospholipids (PLs), glycolipids (GLs), and betaine lipids, have also been recognized as excellent anti-inflammatory mediators [81,82]. In fact, most of the studies mentioned in the previous chapter assessed the anti-inflammatory potential of microalgae polar lipids in comparison to crude extracts and other lipids (Supplementary Table S1). Marine phospholipids are reported as potent anti-inflammatory compounds [16]. The published works described above highlighted (Supplementary Table S1) the anti-inflammatory potential of phospholipid species from microalgae, for example, LPC(16:0) from *Cylindrotheca closterium*, which was capable of inhibiting TNF-α release in pro-inflammatory THP-1 cells [73], or phospholipid fractions from *Spirulina* and *Chlorococcum* sp. SABC 012504, which showed potent capabilities to inhibit PAF and thrombin-induced coagulation [32,33]. Marine phospholipids represent another important source of ω-3 FA and are considered as better deliverers when compared to triglycerides and their free forms. These lipids show more efficient incorporation into tissue membranes at much lower doses, thus contributing to the availability for this ω-3 as a precursor for anti-inflammatory mediators. [16,81]. Additionally, some PLs are also considered inflammatory mediators (e.g., oxidized PLs, plasmalogens), highlighting the phospholipid species itself as an anti-inflammatory component. However, little is known about the relationship between most phospholipid structures and bioactivity (i.e., anti-inflammatory activity), with further research being of extreme importance.

Most of the studies reported above focused their anti-inflammatory screening on microalgae GLs, that have started being recognized recently as potent modulators of different biological activities [17]. These GLs mainly belong to the classes of MGDG, DGDG, and SQDG (Supplementary Table S1) and are another vital source of ω-3 FA. Microalgal GLs were suggested to increase the bioavailability of these PUFAs when compared to triglycerides and their free forms [68]. However, further research is needed to confirm ω-3 FA bioaccessibility from GLs. The glycolipid itself could also be responsible for the observed anti-inflammatory activity. However, the relationship between structure and bioactivity is not fully understood.

**Figure 4:**
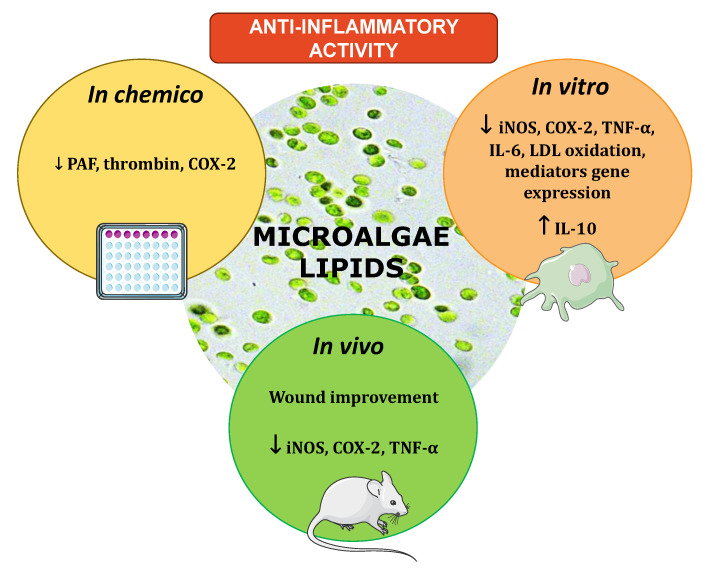
Schematic representation of the anti-inflammatory potential of microalgae lipids. The scheme was based on the data collected and overviewed in Section 3. Microalgae crude lipid extracts, glycolipids, phospholipids, and other isolated lipids (e.g., oxylipins) displayed in vivo and in vitro anti-inflammatory activity with down-regulation of the pro-inflammatory mediators COX-2, iNOS, TNF-α, IL-6, and PAF.

Most of the studies evaluating the reduction of pro-inflammatory mediators (e.g., COX-2, iNOS, TNF-α, or IL-6) [19,50,51,52,53,54,55] were performed in vitro, and very few animal models were used(Figure 4). Clinical studies were also absent. The lack of knowledge towards the complex network of interactions of the inflammatory response still hampers the understanding of the connection between microalgal bioactive lipids and their preventive action against chronic inflammation.

## 5. Concluding Remarks and Future Perspectives

Malnutrition from poor and unhealthy diets increases the prevalence of NCDs and AADs [3]. These conditions are associated with a dysfunctional state of the body systems and subsequent chronic inflammation [7,92]. Over the years, researchers have pointed out the benefits of using diet to attenuate and resolve chronic inflammation and to reduce the risk and prevalence of NCDs and AADs [9]. The dietary consumption of some bioactive lipids (e.g., ω-3 PUFAs) is associated with health benefits, including NCD prevention [93]. Microalgae are rich sources of polar lipids with multiple reported bioactivities [17]. Therefore, this group of microorganisms is used as food and food ingredients, contributing to preventing the development of NCDs and AADs.

Several studies explored the anti-inflammatory potential of microalgae lipids. However, most studies only focused on inhibiting one pro-inflammatory mediator activity/expression, not providing enough evidence for the complex inflammatory mediator networks. Another drawback comes from using different extraction methodologies and solvent systems, hindering the evaluation of the extent of anti-inflammatory potential between crude microalgae extracts. Most studies also lacked investigation surrounding the response of anti-inflammatory mediators in response to microalgae lipids, as they are essential to the transitory response towards the resolution of inflammation. Despite few works exploring the impact of these bioactive compounds in the elements of the inflammatory signaling pathways, there is a lack of evidence regarding the mechanism through which the presented bioactive lipids act.

Despite the strong evidence of microalgae-derived lipids anti-inflammatory activity, the lack of knowledge towards the complex network of interactions of the inflammatory response still hampers the understanding of the connection between microalgal bioactive lipids and their preventive action against chronic inflammation. Further investigations are needed to obtain complete knowledge of the mechanism of action of these lipid products. These studies on their functions as local pro-resolving factors will support the potential use of microalga or derived oxylipins as a therapeutic agent. For that, studies with isolated compounds are needed. Among the bioactive compounds with anti-inflammatory activity, few have entered human clinical trials. Further studies, however, are still required, and they can use in the pre-clinical research from drug development in the near future.

Microalgae are excellent sources of lipid bioactives with potent anti-inflammatory activity and subsequent health benefits. Novel families of oxylipins offer an opportunity as candidates aimed to modulate the inflammatory response. Among these, specialized pro-resolving lipid mediators (SPMs), such as resolvins, protectins, maresins, and lipoxins, are molecules that are highly involved in resolving inflammation. Algae produce these compounds through more alternative pathways than mammals using different enzymes and intermediates but count on similar transformation principles. They inhibit pro-inflammatory transcription factors and activate anti-inflammatory transcription factors [94]. These resolvers biosynthesized from essential ω-3 PUFAs EPA and DHA as precursors are thought to have more potent anti-inflammatory and pro-resolving actions than EPA and DHA themselves [95]. Microalgae produce high amounts ω-3-PUFAs and are therefore promising candidates for producing these specialised resolvers of inflammation. Initial research has revealed that the oily microalgae such as diatoms *Coscinodiscus granii* and *Chaetoceros didymus* produced RvE2 and RvE3 [96].

Regarding the mechanism of inflammation, the disruption of the gut microbiota is closely linked to the pathogenesis of inflammatory diseases. Lipids can act as substrates for bacterial metabolic processes and as inhibitors of bacterial growth by means of toxic effects. A pioneer work has demonstrated that the major fatty acids contained in *Spirulina platensis* 95% ethanol extract helped to regulate gut microbiota in obese and diabetic individuals [97]. Since the gut microbiota can be influenced by dietary lipids, research on the complex interaction between microalgae lipids and the gut microbiota structure and dyslipidemia needs to be addressed in animal models and human cohorts to propose therapeutic strategies. Finally, the bioavailability of lipid species can be significantly dependent on their chemical form. Microalgae have emerged as one of the best alternative sources of bioavailable ω-3 FA and oxylipins. Scientific information regarding the nutritional and phytochemical profiles of the different microalgae found in each geographical site is important. The lack of absorption and bioavailability studies limits the ability of food users to incorporate microalgae in nutrient-based interventions for improving the status of ω-3 PUFAs among the population.

Since inflammation has now been recognized as one of the leading causes of NCDs, the reduction of inflammation by lipids from microalgae can provide beneficial preventative outcomes against several chronic disorders [16,80]. The development of novel nutraceuticals is a possible way of bringing these lipids into the market and making them readily available to consumers and patients. Nevertheless, future research should focus on optimizing microalgae cultivation procedures to increase the yields for such bioactive polar lipids. Therefore, proteomic studies can help determine the biochemical pathways of glycolysis and the synthesis of fatty acids, potentially using excess carbon and nitrogen produced from protein breakdown. Additionally, microalgae cultures aimed to increase algal lipid cell quotas at low cost, combined with optimal growth treatment, could help to optimize lipid production. Additionally, specific food grade and environmentally friendly methods for extracting and separating microalgae polar lipid bioactives need to be investigated and employed according to guidelines for oral consumption in order to further support such health-related nutraceutical applications of microalgae-derived lipid bioactives.

## Supplementary Materials

The following are available online at https://www.mdpi.com/article/10.3390/ijms22189825/s1, Supplementary Table S1: Anti-inflammatory activity of microalgae lipids [19,20,21,22,23,24,25,32,33,50,51,52,56,57,59,60,61,62,63,64,65,66,67,71,72,73,74,75,77,78,79,98].
