# Supplementary material for: Microalgal Lipid Extracts Have Potential to Modulate the Inflammatory Response: A Critical Review
Source: Int J Mol Sci. 2021 Sep 11;22(18):9825. doi: 10.3390/ijms22189825 (PMC8471354; doi:10.3390/ijms22189825)
Supplement: Supplementary file 1 [file ijms-22-09825-s001.zip › ijms-1313769-supplementary.pdf]

Supplementary Table S1—Anti-inflammatory activity of microalgae lipids.

Microalgae species	Lipid	Amount of lipid	Observations	Reference
		Crude Extracts from Microalgae		
Nannochloropsis oculata, Chlorella ovalis, Phaeoductylum tricornutum and *Amphidinium carterae*	Chloroform extracts	6.25-50 µg.mL^{-1}	Suppressed NO production in RAW264.7	[50]
Nannochloropsis oculata	Methanol extracts	6.25-25 µg.mL^{-1}	Downregulated iNOS and COX-2 protein levels	[51]
Gloeothece sp.	Polar lipid extracts	10-1000 µg.mL^{-1}	Inhibited COX-2 enzyme in a COX-2 assay kit	[59]
Chlorella vulgaris auto- and heterotrophically grown	Polar lipid extracts	500 µg.mL^{-1}	Inhibited COX-2 enzyme in a COX-2 assay kit	[19]
Chlorococcum amblystomatis	Polar lipid extracts	50-500 µg.mL^{-1}	Inhibited COX-2 enzyme in a COX-2 assay kit	[61]
Nitzschia palea	Hexane extract	20-60 µg.mL^{-1}	Inhibited COX-2 enzyme in a COX-2 assay kit. Inhibited LPS-induced NO, TNF-α, IL-6 and PGE2 production in Raw264.7.	[60]
Chlorococcum strain SABC0	Chloroform:methanol extracts	-	Inhibited human platelet aggregation induced by PAF and thrombin	[33]
Spirulina	Chloroform:methanol extracts	-	Inhibited rabbit platelet aggregation induced by PAF or thrombin	[32]
Chlorella vulgaris	Chloroform, methanol, and ethanol extracts	7-500 µg.mL^{-1}	InhibitedLPS-induced production of NO, PGE2, TNF-α and IL-6 in Raw 264.7 cells	[62]
Tetraselmis suecica	Methanol extracts	6-100 µg.mL^{-1}	Inhibited of NO, TNF-α and IL-6 release in LPS-induced Raw264.7	[66]

Micractinium sp.	Ethanol extracts	0-40 µg.mL^{-1}	Reduced LPS-induction of COX-2, IL6, iNOS, TNF-α and NO release in Raw264.7 cells.	[20]
Chlorella vulgaris	Ethanolic extracts	-	Attenuated LPS-induced inflammation in white blood cells and by decreasing expression of TNF-α, NF-KB, iNOS e COX-2	[63]
Aurantiochytrium mangrovei	Ethanolic extracts	-	Reduced LPS-induced levels of IL-6, IL-1β, TNF-α, MCP-1 and GM-CFS in RAW264.7 cells.<br>Increased expression of *Socs3* and *Atf3* genes.	[98]
Phaeodactylum tricornutum	Ethanolic extracts	-	Decreased NO, TNF-a, IL-6 and PGE2 production.<br>Decreased LPS-induced mRNA expression of IL-1B, TNF-a, IL-6 and COX-2.	[64]
Chloromonas reticulata	Ethanolic extracts	0-40 µg.mL^{-1}	Reduced LPS-induced mRNA levels of COX-2, iNOS, IL-6 and TNF-α.	[65]
Spirulina maxima	Ethanolic extracts	0.01-0.1 mg.mL^{-1}	Reduced mRNA levels of TNF-α, IL-6 and IL-1β.	[71]
		Lipid fractions from microalgae		
Porphyridium cruentum	Sulfolipids	25-100 µg.mL^{-1}	Inhibited production of O^- generated by peritoneal leukocytes primed with PMA, and the growth of human colon adenocarcinoma DLD-1	[21]
Chlorococcum strain SABC0	Phospholipid fraction, glycolipid fraction	-	Inhibited human platelet aggregation induced by PAF and thrombin	[33]
Spirulina	SQDG and PC-rich fractions	-	Inhibited rabbit platelet aggregation induced by PAF or thrombin	[32]

Spirulina platensis	Glycolipids rich in gamma linolenic acid	50-500 mg.L^{-1}	Reduced neutrophil gathering in wound region in Zebrafish	[23]
Phormidium sp.	MGDG, DGDG and SQDG	-	Attenuated croton-oil induced oedema in mice.	[24]
Nannochloropsis granulata	MGDGs and DGDGs	20-100 µM	Inhibited NO release in LPS-induced in RAW264.7 cells	[67]
Nannochloropsis granulata	DGTSs	20-100 µM	Inhibited NO release in LPS-induced in RAW264.7 cells	[22]
Chlamydomonas debaryana and *Nannochloropsis gaditana*	Oxylipins	25-100 µM	Inhibited TNF-α release in LPS-stimulated THP-1 cells	[72]
Tetraselmis sp. mutant species and *Skeletonema sp.*	Omega-3 PUFAs	1000 µg.mL^{-1}	Inhibited COX-2 enzyme in a COX-2 assay kit	[52]
Microalgae species non-specified	Omega-3 PUFA	1 mg.g^{-1}	Reduced TCD4+ cells production of IFN-γ, TNF-α and IL-4, and increase of TCD4+ cells production of IL-17A, IL-14 and TGF-β, in db/db and CD1mice.	[57]
Pavlova lutheri	SQDG, MGDG, DGDG	3 µg.mL^{-1}	Inhibited LPS-induced production of IL-6 in THP-1 macrophages. Downregulated the expression of pro-inflammatory genes: *TLR1, TLR2, TLR4, TLR8, TRAF5, TRAF6, TNFSF18, IL6R, IL23, CCR1, CCR4, CCL17, STAT3, MAP3K1*. Inhibited the LPS-induced pro-inflammatory TLR signalling pathways, chemokines and NF-κB	[77]
		Isolated lipids from microalgae		
Tetraselmis chui	MGDG(18:3/16:4) MGDG(18:4/16:4)	20-100 µM	Suppressed NO production through downregulation of iNOS in RAW264.7 cells.	[25]
Nannochloropsis sp.	MGTS(20:5)	10 µg.mL^{-1}	Inhibited macrophages and LDL oxidation through increased expression of PON1 in J-774A macrophages	[78,79]
Cylindrotheca Closterium	LPC(16:0)	3.13-µg.mL^{-1}	Inhibited TNF-α release in LPS-stimulated THP-1 cells	[73]

Dunaliella tertiolecta	Ergosterol and 7-dehydroporiferasterol	0-0.8 $mg.mL^{-1}$	Reduced levels of pro-inflammatory cytokines, TNF-α, IL-1β and IL-6, in PBMC. Raised levels of the anti-inflammatory cytokine IL-10.	[75]
Chlamydomonas debaryana	Oxylipin (13S)-HOTE	0.05-1 $mg.kg^{-1}$	Inhibitedcolonic expression of TNF-α and downregulation of iNOS and COX-2 expression in TNBS-induced colitis rats	[56]
Lobosphaera incisa mutant strain	free and esterified DGLA	0-500 µM	Increased base levels of PGE1 secretion in RAW264.7 cells.<br>Attenuated LPC-induced expression of *iNOS*, *Il6* and *LxR* genes.<br>Decreased LPS-induced production of IL-6, NO and ROS.	[74]
